# First person – Ignacio Simó

**DOI:** 10.1242/dmm.053121

**Published:** 2026-09-18

## Abstract

First Person is a series of interviews with the first authors of a selection of papers published in Disease Models & Mechanisms, helping researchers promote themselves alongside their papers. Ignacio Simó is first author on ‘PolyQ-expanded ataxin-3 is associated with mitochondrial alterations in Machado–Joseph disease’, published in DMM. Ignacio is a postdoc in the lab of Angela Laird at Macquarie University, Sydney, NSW, Australia, investigating mitochondrial dysfunction, cellular metabolism and m6A RNA regulation as drivers of neurodegeneration and potential therapeutic targets.

**Figure:**
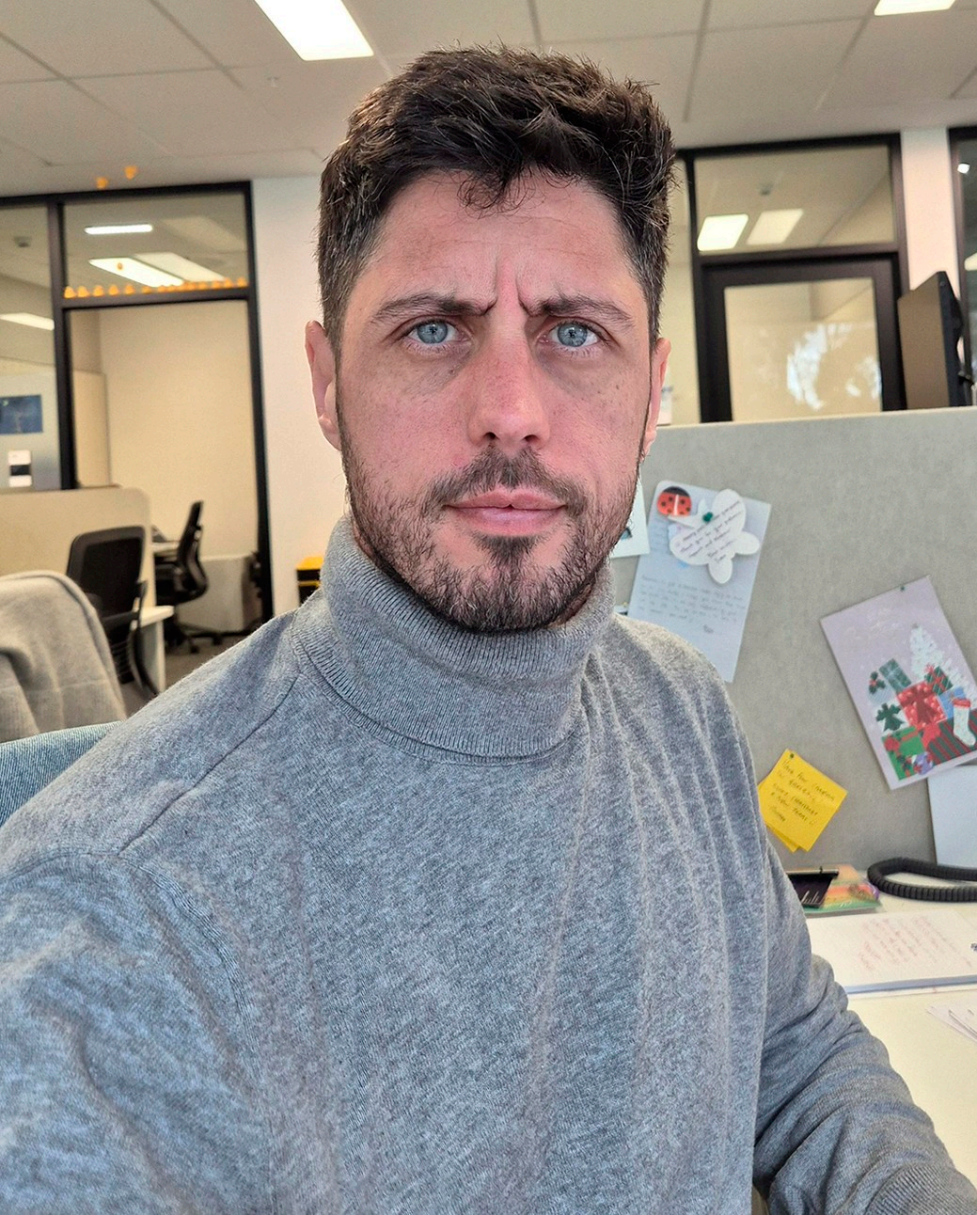
Ignacio Simó

**Who or what inspired you to become a scientist?**

I grew up in a small town in rural Argentina, surrounded by nature. From a very young age, I was fascinated by observing animals and wondering how such complex systems could function in such a coordinated way. That curiosity sparked my interest in biology and eventually led me to pursue a career in science. Although there was not a single scientist who inspired me, my mother played a fundamental role by always encouraging me to follow what I was passionate about and believing in my abilities. Also, when I was a biotechnology undergraduate in Argentina, I joined the Biology Department at the Faculty of Biochemical and Pharmaceutical Sciences at the National University of Rosario as a student assistant, where I was fortunate to have excellent mentors and partners who encouraged me to continue along the research path.

**What is the main question or challenge in disease biology you are addressing in this paper? How did you go about investigating your question or challenge?**

Mitochondrial dysfunction has been reported in Machado–Joseph disease, but it remained unclear whether it represents a consistent feature across different disease models and which molecular mechanisms underlie these defects. In this study, we addressed these questions by examining mitochondrial alterations in complementary models, including transgenic mice, zebrafish and mice primary neurons. We combined proteomic analyses, quantification of electron transport chain complexes, pharmacological inhibition using rotenone, and succinate quantification to investigate the molecular basis of mitochondrial dysfunction. This integrated approach revealed conserved defects across models, including alterations in multiple electron transport chain complexes, with complex II dysfunction and succinate accumulation emerging as key molecular features of the disease.

**How would you explain the main findings of your paper to non-scientific family and friends?**

Think of cells as needing a steady supply of electricity to do their job. They have little ‘power stations’ that keep that electricity flowing. In this disease, we found that those power stations do not run properly, and the important part is that we saw the same kind of problem in different models. We checked in mice, in zebrafish, and in neurons extracted from mice in the lab, and the pattern kept coming back. We also found that the slowdown seems to happen at a particular step in the cell's energy-making process. When that step struggles, it is a bit like a conveyor belt getting jammed: everything behind it starts to pile up. We saw signs of that kind of ‘backup’, which helps explain what is going wrong rather than just saying ‘energy is lower’. So overall, our study says that the energy problem looks like a real, repeatable feature of the disease, and it points to a specific bottleneck that could be a sensible target to focus on in the future.

**What are the potential implications of these results for disease biology and the possible impact on patients?**

These results imply that disrupted cell energy production is likely a core part of the disease process rather than an inconsistent side-effect seen in only one model. Because the same pattern appears across different models, it strengthens the idea that this energy defect is biologically meaningful and may contribute to why certain brain cells become vulnerable over time. They also narrow the mechanism to a specific ‘weak link’ in the energy-making pathway (complex II), alongside a buildup of succinate. Having a clearer bottleneck helps researchers focus on a more precise pathway, instead of treating mitochondrial dysfunction as a broad, vague problem. For patients, the main potential impact is directional: it highlights candidates for biomarkers (metabolic signatures related to succinate/energy pathway changes that could help monitor disease or treatment response) and suggests more targeted therapeutic strategies that aim to correct or bypass that bottleneck. However, these implications still need to be validated in patient-derived samples before they can translate into clinical decisions or treatments.

**Figure:**
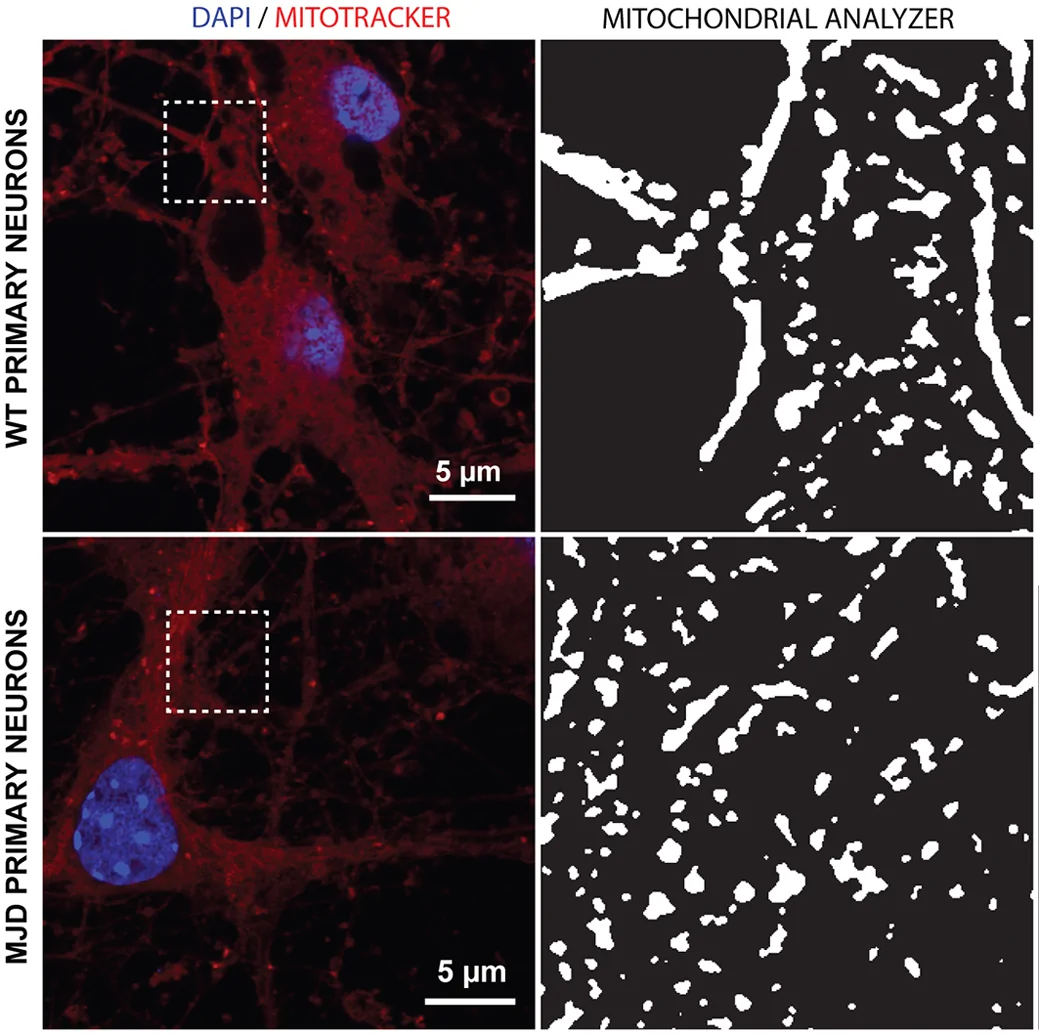
**MitoTracker imaging reveals fragmented mitochondrial networks in primary neurons expressing polyQ ataxin-3.** Combined with molecular analyses, these findings identify widespread electron transport chain alterations, with complex II dysfunction and succinate accumulation emerging as key hallmarks of Machado–Joseph disease.

**Why did you choose DMM for your paper?**

I chose DMM because it is a good home for studies that use disease models to identify the mechanism and are not just descriptive. Our results come from several complementary models and converge on a clear, conserved defect in cell energy pathways, which fits the kind of cross-model, disease-relevant story that DMM publishes. It also reaches the right audience of researchers interested in how these findings could point to future biomarkers or treatment ideas.

**Given your current role, what challenges do you face and what changes could improve the professional lives of other scientists in this role?**

As a postdoc, one of the biggest challenges is the lack of stability that comes with short-term contracts. When appointments are only 1-2 years, it is hard to plan long term, both professionally and personally. A change that would really improve life for postdocs would be a broader, ideally global, shift towards longer baseline contracts (around 4-5 years). With that kind of runway, postdocs could focus properly on the science in front of them, develop stronger ideas and test hypotheses more deeply. Right now, a lot of time and mental energy goes into constantly preparing the next grant or extension, which can pull attention away from doing the careful, creative work that good research needs.

**What's next for you?**

Next for me is continuing to build my profile as an independent researcher in neurodegenerative disease. I am an Argentinian scientist working in Australia and, although my training is in biotechnology and molecular biology, I am learning a lot of neuroscience so I can apply those tools and ways of thinking to understanding and, ultimately, treating neurodegenerative conditions. In the short term, I want to strengthen my expertise at the interface between molecular biology and neuroscience and contribute to projects with clear disease relevance. In parallel, I am aiming to secure competitive funding, with the longer-term goal of establishing my own research line and leading an independent program focused on translating molecular and biotechnological approaches into new therapeutic strategies.

**Tell us something interesting about yourself that wouldn't be on your CV**

As a typical Argentinian with Italian roots, I genuinely love cooking. My specialty is traditional barbecue on the grill, especially the big, all-day kind where a whole pig or lamb is cooked slowly over the fire. I really enjoy the weekend ritual of sharing an asado with family and friends, and it is my way of switching off and recharging when I can step away from the lab for a bit. Honestly, those quiet, relaxed moments are often when the best new ideas in science have room to show up.
